# BLOOD BIOMARKERS OF ALZHEIMER’S DISEASE AND MUSCLE STRENGTH TRAJECTORIES IN COMMUNITY-DWELLING OLDER ADULTS

**DOI:** 10.1093/geroni/igae098.2287

**Published:** 2024-12-31

**Authors:** Alice Ornago, Elena Pinardi, Giulia Grande, Martina Valletta, Amaia Calderón-Larrañaga, Claudia Fredolini, Giuseppe Bellelli, Davide Liborio Vetrano

**Affiliations:** Karolinska Institutet, Osnago, Lombardia, Italy; Karolinska Institutet, Italy; Karolinska Institutet, Stockholm, Stockholms Lan, Sweden; Karolinska Institutet, Stockholm, Stockholms Lan, Sweden; Karolinska Institutet, Stockholm, Stockholms Lan, Sweden; KTH Royal Institute of Technology, Stockholm, Stockholms Lan, Sweden; University of Milano-Bicocca, Milano, Lombardia, Italy; Karolinska Institutet, Stockholm, Stockholms Lan, Sweden

## Abstract

The age-related muscle strength decline is a major impediment to the achievement of healthy aging. This study aimed to investigate the association between a panel of Alzheimer’s disease (AD) blood biomarkers and longitudinal trajectories of muscle strength. This 12-year population-based cohort study included 2011 dementia-free participants from the Swedish National study on Aging and Care in Kungsholmen (SNAC-K). Muscle strength was measured through the grip strength and chair stand tests, while baseline levels of AD biomarkers (total tau [t-tau], phosphorylated tau181 [p-tau181], neurofilament light chain [NfL], glial fibrillary acid protein [GFAP], and amyloid-β [Aβ] 42/40 ratio) were assessed by Quanterix Single Molecule Arrays. Linear mixed models, adjusted for socio-demographics and clinical factors, were used to estimate the association between AD biomarkers and muscle strength decline. Higher baseline levels of p-Tau181 (β 0.94, 95%CI 0.72;1.16), NfL (β 0.79, 95%CI 0.59;0.99), and GFAP (β 0.38, 95% CI 0.22;0.54) were associated with an accelerated worsening of the chair stand test over the follow-up. The adjustment for Mini-Mental State Examination (MMSE) led to an attenuation of these associations. Higher baseline levels of p-Tau181 (β -0.11, 95%CI -0.16;-0.06), and NfL (β -0.05, 95%CI -0.09;-0.003) were associated with a faster decline in the grip strength test over the follow-up; no attenuation was observed after adjusting for MMSE. Several blood biomarkers implicated in AD pathophysiology are associated with distinct trajectories of muscle strength. Cognitive function appears to influence this association, mostly concerning muscle strength as measured by the chair stand test.

